# *MdHB-7* Regulates Water Use Efficiency in Transgenic Apple (*Malus domestica*) Under Long-Term Moderate Water Deficit

**DOI:** 10.3389/fpls.2021.740492

**Published:** 2021-10-28

**Authors:** Shuang Zhao, Hanbing Gao, Xumei Jia, Jiangtong Wei, Ke Mao, Fengwang Ma

**Affiliations:** State Key Laboratory of Crop Stress Biology for Arid Areas/Shaanxi Key Laboratory of Apple, College of Horticulture, Northwest A&F University, Yangling, China

**Keywords:** *MdHB-7*, water use efficiency, stomatal density, root, long-term moderate soil water deficit, *Malus domestica*

## Abstract

Improved water use efficiency (WUE) promotes plant survival and crop yield under water deficit conditions. Although the plant-specific HD-Zip I transcription factors have important roles in plant adaptation to various abiotic stresses, including water deficit, their functions in regulating WUE of apple (*Malus domestica*) are poorly understood. We characterized the role of *MdHB-7* in WUE regulation by subjecting *MdHB-7* transgenic plants to long-term moderate soil water deficit. The long-term WUE (WUE_L_) of transgenic apple plants with *MdHB-7* overexpression or *MdHB-7* RNA interference (RNAi) differed significantly from that of control plants. Upregulation of *MdHB-7* caused reduced stomatal density, whereas the suppression of *MdHB-7* increased stomatal density under both normal and long-term moderate soil water deficit conditions. Moderate reduction in stomatal density helped to improve the WUE of *MdHB-7* overexpression transgenic plants, especially under water deficit conditions. *MdHB-7* overexpression plants maintained high rates of photosynthesis that were conducive to the accumulation of biomass and the improvement of WUE_L_. *MdHB-7* overexpression also alleviated the inhibition of root growth caused by long-term moderate soil water deficit and improved root vitality and hydraulic conductivity, which were essential for improving plant WUE_L_. By contrast, *MdHB-7* RNA interference reduced the WUE_L_ of transgenic plants by inhibiting these factors under normal and long-term moderate soil water deficit conditions. Taken together, our results provide solid evidence for a crucial role of *MdHB-7* in the regulation of apple WUE_L_ and provide new insights for improving the WUE of apple plants under moderate soil water deficit.

## Introduction

Global climate change and the increasing use of groundwater for agriculture have caused severe water shortages for crops in many parts of the world (Shao et al., 2008; Falkenmark, 2013; Wang et al., 2018). Water use efficiency (WUE) is defined as the carbon fixed or biomass produced per unit of water used. It is a comprehensive indicator used to assess plant growth under water deficit. Improvement of WUE is an effective way to reduce production losses due to soil water deficit (Condon et al., 2004; Ali and Talukder, 2008). Apple (*Malus domestica*) is one of the most widely grown and economically important fruits in temperate regions (Zhao et al., 2020b). The Loess Plateau of Shaanxi Province is one of China’s main apple growing regions (Liu et al., 2019). However, as a semi-arid area, the annual rainfall in the Loess Plateau is unevenly distributed. Plants face continuous moderate soil water deficit, which limit the sustainable development of apples in this area (Yan et al., 2015). Therefore, genes related to WUE regulation whose manipulation could improve apple WUE under soil water deficit conditions are important targets for apple breeding.

Many studies have shown the complexity of WUE regulation in plants. The WUE of plants is closely related to two physiological processes, photosynthesis and transpiration, and all factors that affect crop photosynthesis and transpiration have an impact on WUE (Condon et al., 2004; Karaba et al., 2007). Stomata affect WUE by controlling the rates of evapotranspiration and CO_2_ absorption (Yoo et al., 2010; Guo et al., 2019b), and the regulation of stomatal density is a strategy that can improve plant WUE (Yoo et al., 2010; Franks et al., 2015). For example, the overexpression of *EPF* genes in various plant species greatly improved long-term WUE (WUE_L_) by altering stomatal development and density (Franks et al., 2015; Wang et al., 2016; Hughes et al., 2017; Caine et al., 2019). Similarly, the expression of the *Arabidopsis HARDY* gene improved WUE in rice by enhancing photosynthesis and reducing transpiration (Karaba et al., 2007). *MhYTP1* enhanced WUE_L_ of transgenic apple by increasing ABA levels under soil water deficit (Guo et al., 2019a), and *MhYTP2* enhanced WUE_L_ of transgenic apples by activating ABA and ethylene signaling (Liu et al., 2019). Roots are the main interface through which plants absorb water from the surrounding soil, and the root system is therefore considered to be a key determinant of WUE under various levels of soil water deficit (Coudert et al., 2010; Wang et al., 2020).

The homeodomain–leucine zipper (HD-Zip) transcription factor (TF) family is unique to plants and plays an important role in stress response regulation (Schena and Davis, 1992; Ariel et al., 2007; Gong et al., 2019). HD-Zip TFs have been identified in multiple species and divided into four subfamilies (Ariel et al., 2007). Many HD-Zip I members have been shown to function in the adaptive response to abiotic stress. For example, *ATHB7* and *ATHB12* were strongly induced by water deficiency and ABA treatment (Ré et al., 2014). Overexpression of *ATHB13* or *HaHB1* stabilized cell membrane integrity in transgenic *Arabidopsis* and increased plant tolerance of drought and salt stress (Cabello and Chan, 2012). Overexpression of the maize HD-Zip I genes *ZmHDZ4* and *ZmHDZ10* reduced relative electrolyte leakage (REL) and malondialdehyde (MDA) content, thereby conferring drought tolerance on transgenic rice (Zhao et al., 2014; Wu et al., 2016). *TaHDZipI-5* conferred freezing and drought tolerance on transgenic wheat plants (Yang et al., 2018). Overexpression of *HaHB4* improved the water deficit tolerance, yield, and WUE of transgenic soybean plants (Ribichich et al., 2020). Although the roles of several HD-Zip I TFs in the regulation of abiotic stress response and tolerance have been characterized, the roles of most HD-Zip I TFs in regulating WUE remain unclear, especially in woody plants.

In our recent work, we found that the HD-Zip I TF *MdHB-7* positively regulated apple drought tolerance. *MdHB-7* overexpressing (OE) transgenic apple plants were more tolerant of drought treatment, whereas *MdHB-7* RNA interference (RNAi) plants were more sensitive (Zhao et al., 2020a). It is important to note that short-term drought produced by withholding irrigation differs from the long-term moderate soil water deficit that is common during apple production in the Loess Plateau. Furthermore, the increased tolerance of *MdHB-7* transgenic plants to drought does not necessarily mean that the gene positively regulates WUE, especially under long-term moderate soil water deficit. In order to study the effect of *MdHB-7* on WUE and to explore the possible mechanisms by which *MdHB-7* regulates WUE in apple plants, such as affecting stomatal density and root water uptake capacity, we cultivated *MdHB-7* transgenic apple plants under long-term (60days) moderate soil water deficits and evaluated their performance.

## Materials and Methods

### Plant Materials, Growth Conditions, and Treatments

GL-3 (“Royal Gala”) plants were obtained from Dai et al. (2013). *MdHB-7* OE and *MdHB-7* RNAi transgenic lines were generated in our previous studies (Zhao et al., 2020b) and were subcultured according to the method of Sun et al. (2018). GL-3 and *MdHB-7* transgenic plants were rooted as described in Zhou et al. (2019). Rooted GL-3 plants and *MdHB-7* transgenic lines were transplanted into plastic pots filled with nutrient soil, vermiculite, and perlite (3:1:1; v:v:v), then grown in an artificial climate chamber under a 16/8-h light/dark photoperiod at a temperature of 23–25°C. After 1month of adaptation, plants of similar size were transplanted into pots (38cm×23cm) filled with loess soil, sand, and organic matter (5:1:1; v:v:v) and grown in the greenhouse of Northwest A & F University in Yangling (34°20'N, 108°24'E), Shaanxi Province, China. The weight of soil in each pot was 13.5±0.1kg. When the plants had grown to about 60cm in height, they were divided into a well-watered control group and a moderate soil water deficit treatment group. Forty plants were used from each genotype and were divided into two groups, one group for sampling and one group for the final biomass statistics. Seedlings were irrigated so that the control pots were maintained at 75–85% of maximum field capacity and the soil water deficit-treated pots were maintained at 45–55% of maximum field capacity (Geng et al., 2018). Maximum water field capacity was defined as (W1−W2)/W2, where W1 is the saturated soil weight, and W2 is the dry soil weight. The soil texture and weight of all pots were the same, so the maximum field capacity of all pots was also the same. Therefore, the weight of each pot at 75–85 or 45–55% of maximum field capacity could be calculated. GL-3 and transgenic plants were irrigated every 2days. And, all pots were weighed before each watering in order to calculate the amount of water to be added, and this amount was recorded. At the end of the experiments, the total water consumption was calculated.

### Physiological Analyses

All photosynthetic measurements were obtained using a Li-6,400 portable photosynthesis system (Li-Cor, Inc., Lincoln, NE, United States) with 1,000μmol photons m^−2^ s^−1^ and a cuvette CO_2_ concentration of 400μmol CO_2_ mol^−1^ air. Net photosynthetic rate (Pn), stomatal conductance (gs), transpiration rate, and instantaneous WUE (WUEi) were measured on at least five plants from each genotype. Chlorophyll content, leaf relative water content, and water loss were measured as described by Hu et al. (2018) and Jiang et al. (2019).

The triphenyltetrazolium chloride (TTC) method was used to assess the effect of long-term soil water deficit on the root activity of GL-3 and *MdHB-7* transgenic plants (Huo et al., 2020). Root hydraulic conductance was measured using a pressure chamber (Model 1505D, PMS Instrument Company, Albany, OR, United States) as described by Zhu et al. (2015). At the end of the experiment, the roots of the apple plants were too large to directly measure the root hydraulic conductivity at the whole-root level. For each plant, five similarly sized lateral roots were selected for the measurement of root hydraulic conductivity. The average root hydraulic conductivity value of the five lateral roots was used to represent the root hydraulic conductivity of the plant. For each genotype, six plants were randomly selected for measurement of root hydraulic conductivity.

The plant height and stem diameter of GL-3 and *MdHB-7* transgenic plants were measured using a tape measure and vernier caliper, respectively.

### Biomass Accumulation and Long-Term Water Use Efficiency

At the beginning and end of the moderate soil water deficit treatment, the dry weights of roots, stems, and leaves of GL-3 and transgenic plants were measured. At the end of the experiment, 18 plants from each line that had not previously been sampled during the experiment were used for biomass statistics. The relative growth rate (RGR) was calculated as described previously (Radford, 1967). RGR=(Ln DW2−Ln DW1)/(T2−T1). DW2 is the plant dry weight at the final harvest time (T2) of the moderate soil water deficit treatment, and DW1 is the plant dry weight at the initial time (T1) of the moderate soil water deficit treatment. Long-term water use efficiency (WUE_L_) was calculated as the ratio of the accumulation of total dry mass produced to total water used (Ehdaie and Waines, 1993). WUE_L_=(DW2−DW1)/total water consumption.

### Stomatal Characteristics

Leaf stomata were observed under an EX30 microscope (SDPTOP). At least 10 fully expanded leaves from the same stem position were harvested from each genotype after 60days of moderate soil water deficit. The number of stomata in each image was recorded using Image J software and used to calculate the final stomatal density.

### RNA Extraction and qRT-PCR Analysis

Total RNA was extracted from leaves using the Plant RNA Isolation Kit from Wolact [Wolact, Vicband Life Sciences Company (Hk) Limited]. First-strand cDNA was synthesized using a RevertAid First Strand cDNA Synthesis Kit (Thermo Scientific), and the reaction products were diluted to 100ngμl^−1^ with sterile water. Real time qRT-PCR analysis was performed as previously described by Zhao et al. (2020b), and all the primers used are listed in Supplementary Table S1.

### Statistical Analysis

SPSS Version 17.0 (SPSS Inc., Chicago, IL, United States) was used for statistical analysis. Data were analyzed by one-way ANOVA followed by Tukey’s multiple range test, and experimental data were presented as mean±SD. Differences were considered as statistically significant at *p*<0.05.

## Results

### *MdHB-7* Promotes Plant Growth Under Long-Term Moderate Soil Water Deficit

**Figure 1:**
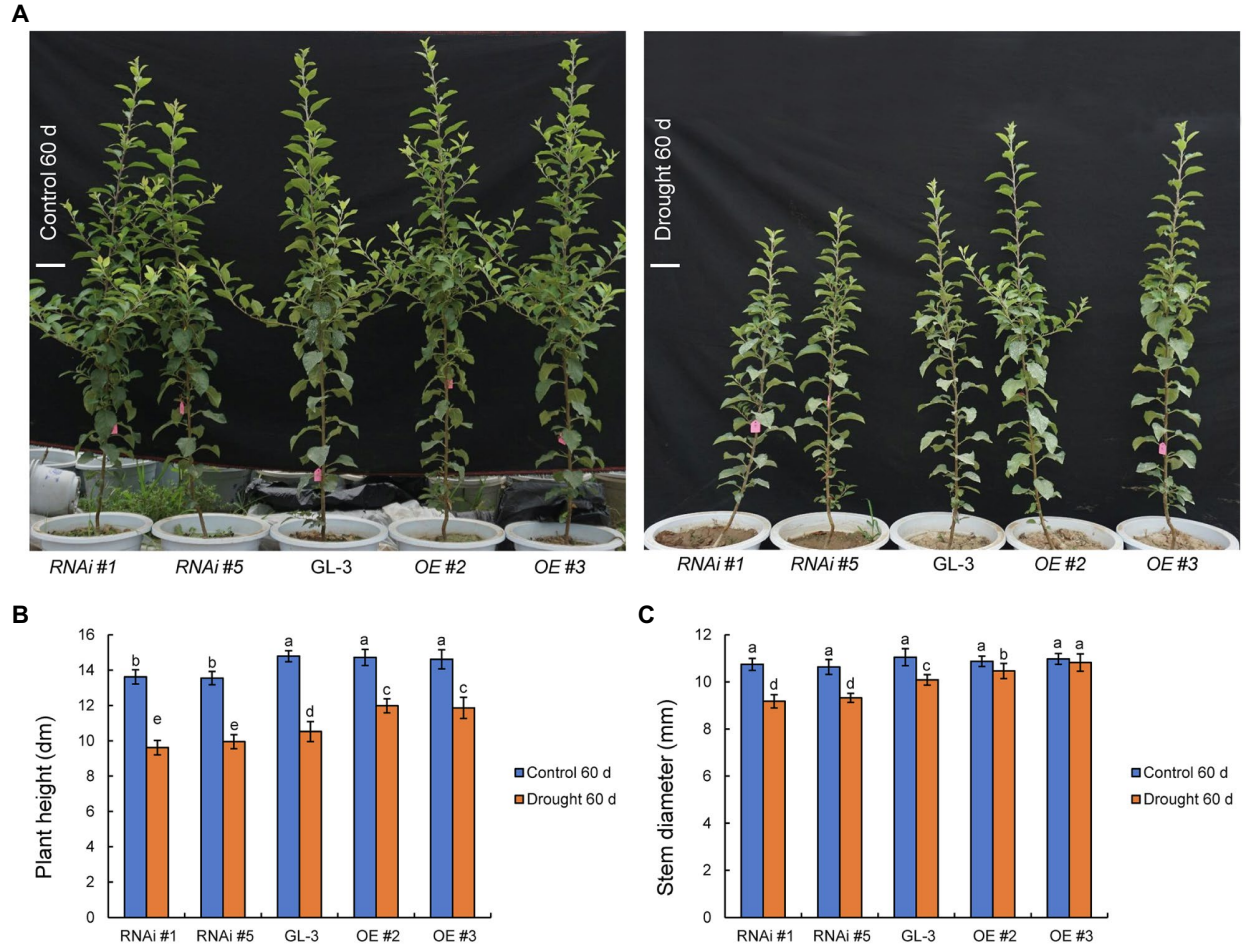
Comparison of the growth status of GL-3 and transgenic apple plants under long-term moderate soil water deficit. **(A)** Phenotypic comparison of GL-3 and *MdHB-7* transgenic apple plants grown in well-watered and moderate soil water deficit conditions for 60days. RNAi and OE represent the *MdHB-7*-RNAi and *MdHB-7*-overexpressing apple plants, respectively. Scale bars, 10cm. **(B)** Plant height. **(C)** Stem diameter. Data are means±SD (*n*=18 for **B**,**C**; 18 plants from each line). Different letters indicate significant differences between GL-3 and transgenic apple plants based on one-way ANOVA and Tukey’s multiple range test (*p*<0.05).

At the beginning of the experiment, there were no significant differences in plant height, stem diameter, and dry weight between GL-3 and *MdHB-7* transgenic plants (Supplementary Figure S1). Under well-watered conditions, the plant height and stem diameter of GL-3 and *MdHB-7* OE lines (OE #2 and OE #3) showed no significant difference throughout the experimental period (Figure 1; Supplementary Figure S2). The height of *MdHB-7* RNAi lines was significantly lower than that of the GL-3 and *MdHB-7* overexpression lines after more than 40days of growth under normal conditions (Figure 1; Supplementary Figure S2). Sixty days of long-term moderate soil water deficit inhibited the growth of GL-3 and transgenic plants compared with the control group. At increasing treatment durations, the growth of the *MdHB-7* overexpression transgenic lines gradually became better than that of the GL-3 and RNAi plants (Figure 1; Supplementary Figure S2). Compared with GL-3 plants, *MdHB-7* OE lines had taller shoots and thicker stems under moderate soil water deficit, whereas *MdHB-7* RNAi lines had shorter shoots and thinner stems (Figures 1A–C). To eliminate the possibility that *MdHB-7* affected plant growth by regulating the expression of other *HD-Zip* genes, we examined the expression of *MdHD-Zips* in GL-3 and *MdHB-7* transgenic plants. These *MdHD*-*Zips* have high sequence similarity to *MdHB-7*, and their expression was not significantly altered in *MdHB-7* transgenic plants (Supplementary Figure S3). These results indicated that the weak growth of *MdHB-7* RNAi plants was due directly to *MdHB-7* suppression, rather than the influence of *MdHB-7* on other *MdHD-Zips*. *MdHB-7* therefore had a positive effect on plant growth under long-term moderate soil water deficit.

### *MdHB-7* Regulates Biomass Accumulation, RGR, and WUE_L_ Under Long-Term Moderate Soil Water Deficit

**Figure 2:**
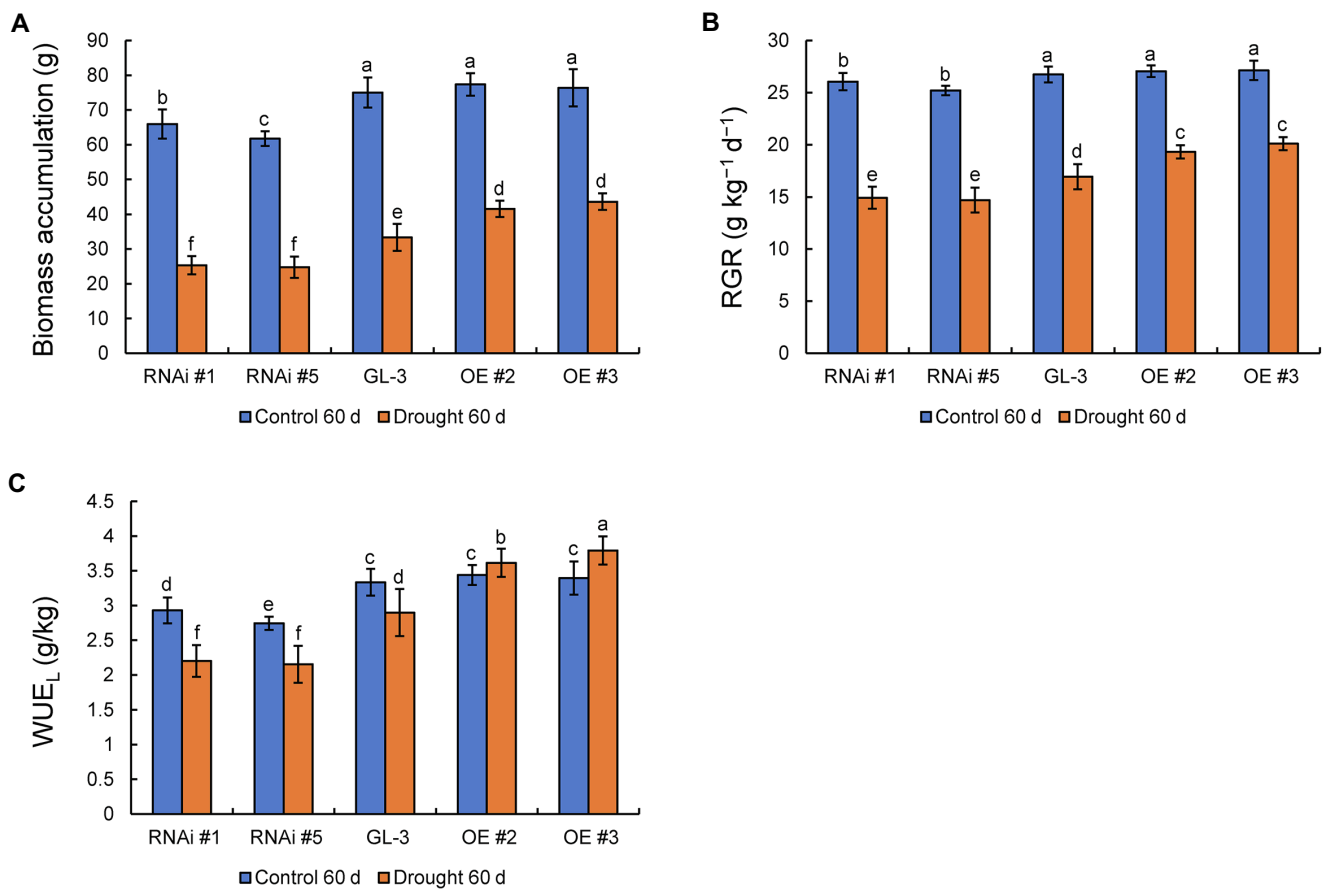
Biomass accumulation, RGR, and long-term WUE (WUE_L_) of GL-3 and *MdHB-7* transgenic plants after 0 and 60days under moderate soil water deficit conditions. **(A)** Biomass (plant dry weight). **(B)** Relative growth rate (RGR). **(C)** WUE_L_. Data are means±SD (*n*=18; 18 plants from each line). Different letters indicate significant differences between GL-3 and transgenic apple plants based on one-way ANOVA and Tukey’s multiple range test (*p*<0.05).

There were no significant differences in biomass accumulation and RGR between GL-3 and *MdHB-7* OE lines under normal conditions, whereas the *MdHB-7* RNAi lines accumulated significantly lower biomass and had a lower RGR. Compared with the control group, moderate soil water deficit significantly inhibited the biomass accumulation and RGR of GL-3 and *MdHB-7* transgenic plants. Under long-term moderate soil water deficit, *MdHB-7* OE lines accumulated more biomass and had higher RGR than GL-3, whereas *MdHB-7* RNAi lines accumulated less biomass and had the lowest RGR (Figures 2A,B). There were no differences in WUE_L_ between GL-3 and *MdHB-7* OE lines after 60days under normal conditions. Under long-term moderate soil water deficit, the WUE_L_ of *MdHB-7* OE lines was significantly higher than that of GL-3. The WUE_L_ of the RNAi lines was lower than that of GL-3 under both normal and drought conditions (Figure 2C). Interestingly, compared with normal conditions, moderate soil water deficit inhibited the WUE_L_ of GL-3 and RNAi lines but increased the WUE_L_ of OE plants (Figure 2C). These results indicated that *MdHB-7* promoted biomass accumulation and RGR and improved WUE_L_ under long-term moderate soil water deficit.

### *MdHB-7* Affects Stomatal Density

**Figure 3:**
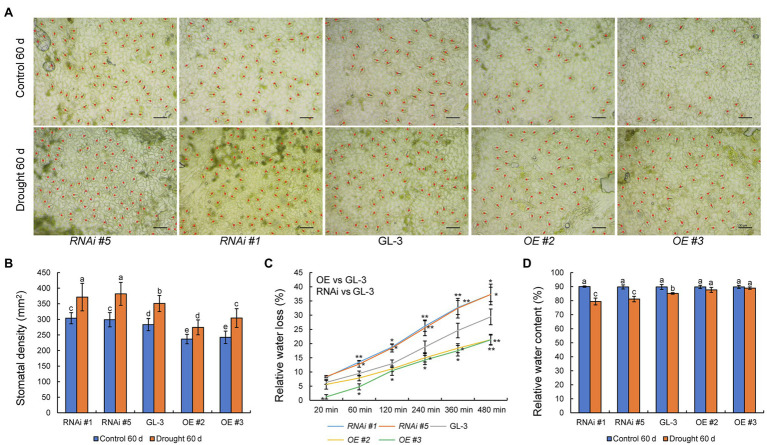
Effect of *MdHB-7* expression on stomatal density, leaf water loss, and relative water content. **(A)** Micrographs of abaxial leaf epidermis from GL-3 and *MdHB-7* transgenic plants. Scale bars, 50μm. **(B)** Stomatal density of the leaf abaxial epidermis of GL-3 and *MdHB-7* transgenic plants. **(C)** Relative water loss from detached leaves measured at the indicated time points. **(D)** Relative water content. Data are means±SD (*n*≥50 for **B**, at least 50 stomatal densities were measured per genotype; *n*=5 for **C**,**D**). Different letters indicate significant differences among genotypes based on one-way ANOVA and Tukey’s multiple range test (*p*<0.05).

Stomatal density plays an important role in the regulation of WUE, and members of the HD-Zip family are known to regulate plant stomatal density (Mishra et al., 2012). We therefore examined the stomatal density of GL-3 and *MdHB-7* transgenic plants. Under normal conditions, stomatal density was lower in *MdHB-7* OE lines and higher in *MdHB-7* RNAi lines than in GL-3 plants (Figures 3A,B). Long-term moderate soil water deficit increased the stomatal density of all genotypes. Nonetheless, the stomatal density of OE lines was still lower than that of GL-3 plants, and the stomatal density of RNAi lines was higher. Stomatal density affects leaf water loss, which in turn affects plant adaptability to water deficit and WUE (Jiang et al., 2019). We found that relative leaf water loss was lower in *MdHB-7* OE lines than in GL-3, whereas *MdHB-7* RNAi lines had the highest relative water loss (Figure 3C). Changes in stomatal density also influence leaf relative water content (Yoo et al., 2010), thereby affecting the adaptability of plants to water deficit. After 60days of long-term moderate soil water deficit, the relative water content was higher in *MdHB-7* OE lines than in GL-3, whereas the relative water content of *MdHB-7* RNAi lines was lower (Figure 3D). These results suggest that *MdHB-7* inhibits water loss under soil water deficit conditions by reducing stomatal density, thereby improving WUE_L_.

**Figure 4:**
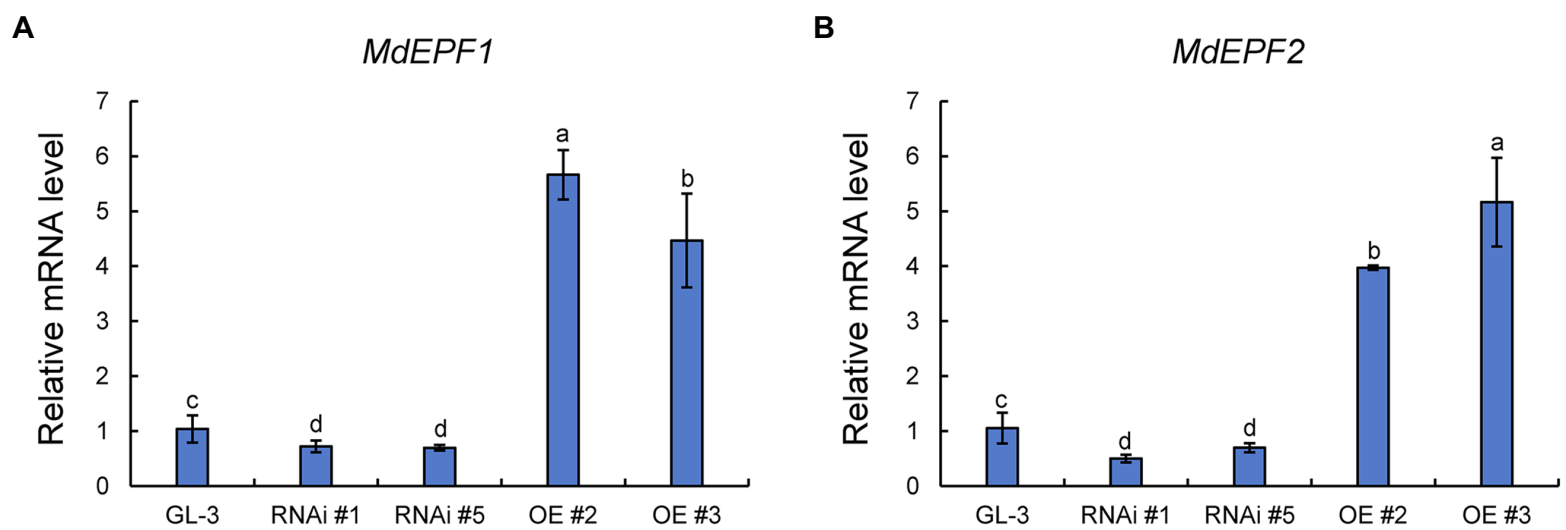
Relative expression of *MdEPF1* **(A)** and *MdEPF2* **(B)** in the leaves of GL-3 and *MdHB-7* transgenic plants. Data are presented as means±SD (*n*=3). Different letters indicate significant differences based on one-way ANOVA and Tukey’s multiple range tests (*p*<0.05).

The epidermal patterning factors (EPF) encode a secreted peptide family (EPF1 and EPF2) that play a vital role in stomatal development process (Wang et al., 2016). The ectopic expression of *MdEPF2* in tomato reduced the stomatal density of transgenic plants (Jiang et al., 2019). As shown in Figure 4, the expression levels of *MdEPF1* and *MdEPF2* were higher in *MdHB-7* OE lines than in GL-3, and their expression was lower in *MdHB-7* RNAi lines. These results suggested that the effect of *MdHB-7* on stomatal density may depend on its direct or indirect regulation of *MdEPF1* and *MdEPF2*.

### *MdHB-7* Affects Photosynthetic Rate and WUEi Under Long-Term Moderate Soil Water Deficit

**Figure 5:**
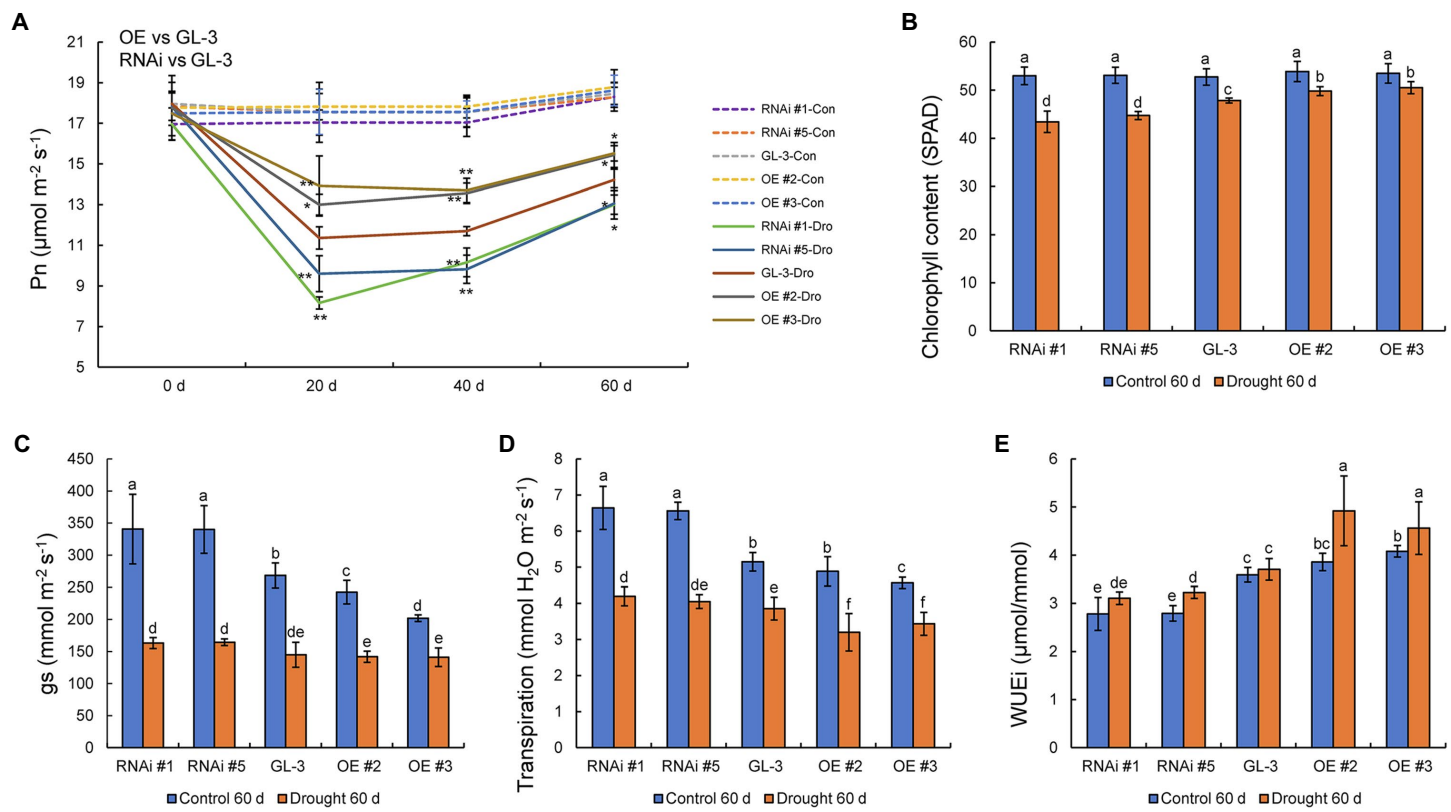
Physiological indices of GL-3 and *MdHB-7* transgenic apple plants under well-watered and soil water deficit conditions. **(A)** Photosynthetic rate (Pn). **(B)** Chlorophyll content. **(C)** Stomatal conductance (gs). **(D)** Transpiration. **(E)** WUEi. Data are means±SD (*n*=5 for **A–E**). In comparison with GL-3, ^*^p<0.05 and ^**^p<0.01. Different letters indicate significant differences between lines based on one-way ANOVA and Tukey’s multiple range test (*p*<0.05).

Water deficits affect photosynthetic efficiency, and we found that the Pn of all genotypes decreased under long-term moderate soil water deficit. The decline in Pn under moderate soil water deficit was lowest in the *MdHB-7* OE lines and greatest in the RNAi lines. There were no significant differences in Pn between GL-3 and *MdHB-7* transgenic plants under well-watered conditions (Figure 5A). The efficiency of photosynthesis is closely related to chlorophyll accumulation, and we therefore measured the chlorophyll content of all genotypes. Chlorophyll content was reduced in all lines under long-term moderate soil water deficit, but the chlorophyll content of *MdHB-7* OE lines decreased less than that of GL-3 and RNAi plants (Figure 5B).

Stomatal density affects plant transpiration rate and leaf gas exchange, which are crucial determinants of photosynthesis. Under normal conditions, values of gs were significantly lower in *MdHB-7* OE lines than in GL-3 and RNAi lines. Long-term moderate soil water deficit reduced gs values in GL-3 and transgenic plants. Under long-term moderate soil water deficit, gs values were slightly lower in *MdHB-7* OE lines than in GL-3, and gs values were higher in RNAi lines than in GL-3, but there were no significant differences (Figure 5C). Under well-watered conditions, the transpiration rate was significantly lower in OE #3 and slightly lower in OE #2 compared with GL-3. By contrast, the transpiration rate of the RNAi lines was significantly higher than that of GL-3 (Figure 5D). Under long-term moderate soil water deficit, the transpiration rate was significantly lower in the two OE lines than in GL-3. The transpiration rate of RNAi #1 was significantly higher than that of GL-3; that of RNAi #5 was also higher, but this difference was not significant (Figure 5D). Under well-watered and long-term moderate soil water deficit, WUEi was higher in *MdHB-7* OE lines and lower in RNAi lines compared with GL-3 (Figure 5E). Interestingly, the WUEi of OE plants under long-term moderate soil water deficit was significantly higher than that of OE plants under normal conditions (Figure 5E). These results suggest that the overexpression of *MdHB-7* enhanced the photosynthetic ability and WUEi of plants under long-term moderate soil water deficit.

### *MdHB-7* Affects Root Activity and Hydraulic Conductivity Under Long-Term Moderate Soil Water Deficit

**Figure 6:**
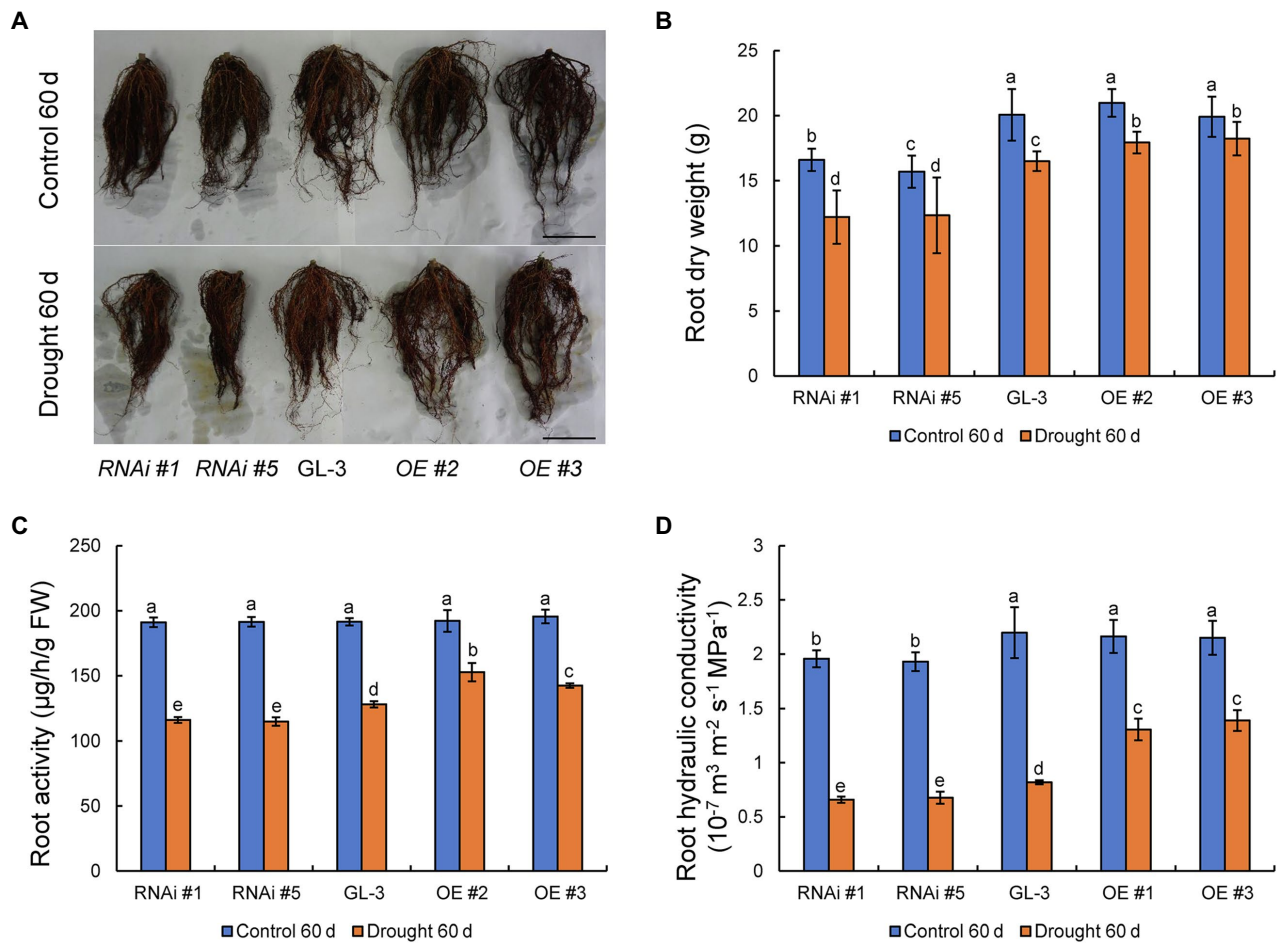
Root growth and physiology of GL-3 and *MdHB-7* transgenic apple plants under long-term moderate soil water deficit. **(A)** Root morphology. Scale bars, 5cm. **(B)** Root dry weight. **(C)** Root activity measured by the TTC method. **(D)** Root hydraulic conductivity. Data are expressed as means±SD (*n*=18 for B, 18 plants from each line; *n*=3 for C, three biological replicates; *n*=6 for D, six plants from each line). Within a sampling date, different letters indicate significant differences based on one-way ANOVA and Tukey’s multiple range test (*p*<0.05).

In addition to aboveground plant parts, the growth of plant roots and their absorption and transport of water also influence WUE under moderate soil water deficit conditions. We previously demonstrated that *MdHB-7* was highly expressed in apple roots (Zhao et al., 2020a). Here, we examined the roots of GL-3 and *MdHB-7* transgenic plants under normal and long-term moderate soil water deficit conditions. Under normal conditions, there were no differences in root growth and dry weight between GL-3 and *MdHB-7* OE lines. By contrast, root growth and dry weight were significantly lower in *MdHB-7* RNAi lines than in GL-3 (Figures 6A,B). Root growth of all genotypes was inhibited by long-term moderate soil water deficit. Nonetheless, the root dry weights of *MdHB-7* OE and RNAi lines were significantly higher and lower, respectively, than those of GL-3 (Figures 6A,B). Likewise, root activity was higher in *MdHB-7* OE lines and lower in *MdHB-7* RNAi lines under moderate soil water deficit (Figure 6C). Root hydraulic conductivity decreased significantly in all genotypes under long-term moderate soil water deficit, but it was higher in *MdHB-7* OE lines and lower in RNAi lines compared with GL-3 (Figure 6D). These results indicated that *MdHB-7* overexpression promotes the root growth and helps to maintain greater water transport capacity in transgenic apple plants under long-term moderate soil water deficit.

## Discussion

Stomata are two-celled valves that control epidermal pores and play a central role in leaf transpiration and CO_2_ absorption (Le et al., 2014; Lee et al., 2017). Here, we showed that the overexpression of *MdHB-7* reduced stomatal density (Figures 3A,B) and decreased transpiration rate and water loss rate (Figures 3C, 5D). The decrease in stomatal density decreases leaf water loss and increases leaf relative water content (Yoo et al., 2010; Jiang et al., 2019). Interestingly, the difference between the relative water content of leaves of each genotype after long-term moderate drought treatment was not as large as the difference in relative water loss of isolated leaves of each genotype. It might be that the root system of the plant was able to continuously absorb water to replenish the leaves during the long-term moderate drought treatment, while the isolated leaves were not supplied with water. Moreover, the reduced stomatal density caused by *MdHB-7* overexpression did not affect CO_2_ assimilation and biomass accumulation and increased WUE. Previous studies have shown that the net CO_2_ assimilation rate reached saturation with increasing stomatal conductance, while the increase in transpiration rate remained linear (Yoo et al., 2009). Therefore, a moderate reduction in stomatal density can significantly reduce the transpiration rate and leaf water loss without simultaneously affecting CO_2_ assimilation and improving WUE (Yoo et al., 2010; Guo et al., 2019b; Li et al., 2020). Previous studies have shown that long-term moderate drought treatment increased stomatal density in newly developed leaves of apple plants (Liang et al., 2018a,b; Jia et al., 2021). The leaf area of the plant decreased after long-term moderate drought treatment, which might be responsible for the increase in stomatal density (Jiang et al., 2019). Besides, altering the stomatal density of plants is a strategy for plants to adapt to long-term moderate water deficit (Li et al., 2020; Jia et al., 2021).

Stomatal density is influenced by stomatal development (Xiang et al., 2021). Peptides play an important role in stomatal development. EPF1 and EPF2 belong to the EPF family of secretory peptides and participate in multiple aspects of stomatal development (Wang et al., 2016). Overexpression of *PdEPF1* has been shown to reduce stomatal density on the back of the leaf, thereby reducing transpiration, maintaining leaf relative water content, and enhancing WUE_L_ (Wang et al., 2016). The leaves of *AtEPF2* OE *Arabidopsis* plants showed significantly lower stomatal density and greater WUEi and WUE_L_, whereas *epf1epf2* mutants exhibited higher stomatal density and lower WUEi and WUE_L_ (Franks et al., 2015). Here, the expression of *MdEPF1* and *MdEPF2* was higher in *MdHB-7* OE lines and lower in *MdHB-7* RNAi lines (Figure 4). This result, combined with the fact that overexpression of *MdEPF2* leads to reduced stomatal density in transgenic plants and improved WUE_L_ under long-term water deficit (Jiang et al., 2019), suggests that *MdHB-7* may affect stomatal density by influencing the expression of *EPF* family genes, such as *MdEPF1* and *MdEPF2*, and thus WUE_L_. Further studies are needed to verify this regulatory mechanism, including studies on the direct or indirect regulatory effect of *MdHB-7* on the expression of EPF family genes.

The maintenance of a high photosynthetic rate is important for improving WUE (Karaba et al., 2007; Condon, 2020), especially under stress conditions. The regulation of photosynthesis contributed significantly to higher WUE_L_ in apple plants under long-term moderate water deficit (Zhou et al., 2015). Here, the overexpression of *MdHB-7* improved WUE_L_ partly by maintaining a high photosynthetic rate under long-term moderate soil water deficit (Figure 5A). Photosynthesis is affected by multiple mechanisms, including stomatal restriction, and water deficit leads to a decrease in stomatal conductance (Warren et al., 2011). Another mechanism is the limitation associated with non-stomatal factors, such as decreases in leaf chlorophyll content (Pagter et al., 2005). Under long-term moderate soil water deficit, there was no significant difference in gs between *MdHB-7* transgenic lines and GL-3 plants (Figure 5C). Therefore, we speculated that Pn may have been limited primarily by non-stomatal factors such as ribulose 1,5-bisphosphate regeneration and chlorophyll content. Chlorophyll is the main photosynthetic pigment and has an important role in light absorption, transmission, and transformation (Zuo et al., 2014). If chlorophyll levels are reduced, the absorption of light energy by the chloroplast will also be reduced (Mafakheri et al., 2010). Water deficit induces the production of reactive oxygen species in leaves and promotes the degradation of chlorophyll (Liang et al., 2018b). The chlorophyll content of all genotypes decreased under long-term moderate soil water deficit, but that of *MdHB-7* OE lines decreased least (Figure 5B), allowing them to maintain a higher Pn (Figure 5A). These results indicated that the overexpression of *MdHB-7* could help to maintain high chlorophyll content in apple leaves under long-term moderate soil water deficit, thereby enabling *MdHB-7* OE lines to maintain a higher Pn, which in turn promoted biomass accumulation and improved WUE_L_.

Roots play a key role in water and nutrient absorption and in sensing dehydration stress signals and transferring them to shoots. Therefore, roots are the key to solving problems caused by water shortage (Wang et al., 2020). Root vitality refers to the absorption, synthesis, oxidation, and reduction capabilities of the root system; it can be used as a physiological indicator to objectively quantify root system activity (Lin and Fan, 2013). The ability of the root system to transport water from the surrounding soil can be evaluated by the root hydraulic conductivity, and higher root hydraulic conductivity usually indicates a greater potential for water transfer from soil to roots (Geng et al., 2018; Jia et al., 2020). Overexpression of *MdMYB88* or *MdMYB124* positively regulated root architecture and increased root hydraulic conductivity of transgenic apple plants relative to GL-3 under long-term moderate soil water deficit, promoting greater adaptation of transgenic plants to moderate water deficit (Geng et al., 2018). Our results indicated that the overexpression of *MdHB-7* alleviated the inhibition of root growth caused by long-term moderate soil water deficit. Compared with GL-3, *MdHB-7* OE plants had greater root vitality and hydraulic conductivity under long-term moderate soil water deficit (Figure 6), and this may explain why *MdHB-7* overexpression improved WUE_L_.

In conclusion, overexpression of *MdHB-7* improved WUE_L_ under long-term moderate soil water deficit by reducing stomatal density and water loss and promoting high photosynthetic rates. Overexpression of *MdHB-7* also minimized the root growth inhibition caused by long-term moderate soil water deficit and increased root vitality and hydraulic conductivity. Our findings provide new evidence for a role of HD-Zip TFs in improving the WUE_L_ of apple plants under long-term moderate soil water deficit.

## Data Availability Statement

The original contributions presented in the study are included in the article/Supplementary Material; further inquiries can be directed to the corresponding author.

## Author Contributions

FM, KM, and SZ conceived and designed the study. SZ, HG, XJ, and JW performed the analyses. SZ drafted the manuscript. FM and KM supervised the process of this research and provided financial support for the study. All authors contributed to the article and approved the submitted version.

## Funding

This work was supported by the National Key Research and Development Program of China (2018YFD1000303), the National Natural Science Foundation of China (31972391), and the earmarked fund for the China Agricultural Research System (CARS-27).

## Conflict of Interest

The authors declare that the research was conducted in the absence of any commercial or financial relationships that could be construed as a potential conflict of interest.

## Publisher’s Note

All claims expressed in this article are solely those of the authors and do not necessarily represent those of their affiliated organizations, or those of the publisher, the editors and the reviewers. Any product that may be evaluated in this article, or claim that may be made by its manufacturer, is not guaranteed or endorsed by the publisher.
