# Supplementary material for: MdHB-7 Regulates Water Use Efficiency in Transgenic Apple (Malus domestica) Under Long-Term Moderate Water Deficit
Source: Front Plant Sci. 2021 Oct 28;12:740492. doi: 10.3389/fpls.2021.740492 (PMC8582324; doi:10.3389/fpls.2021.740492)
Supplement: Supplementary file 1 [file Data_Sheet_1.docx]

**Figure S1.** GL-3 and *MdHB-7* transgenic apple plants subjected to long-term moderate water deficit for 0 d.


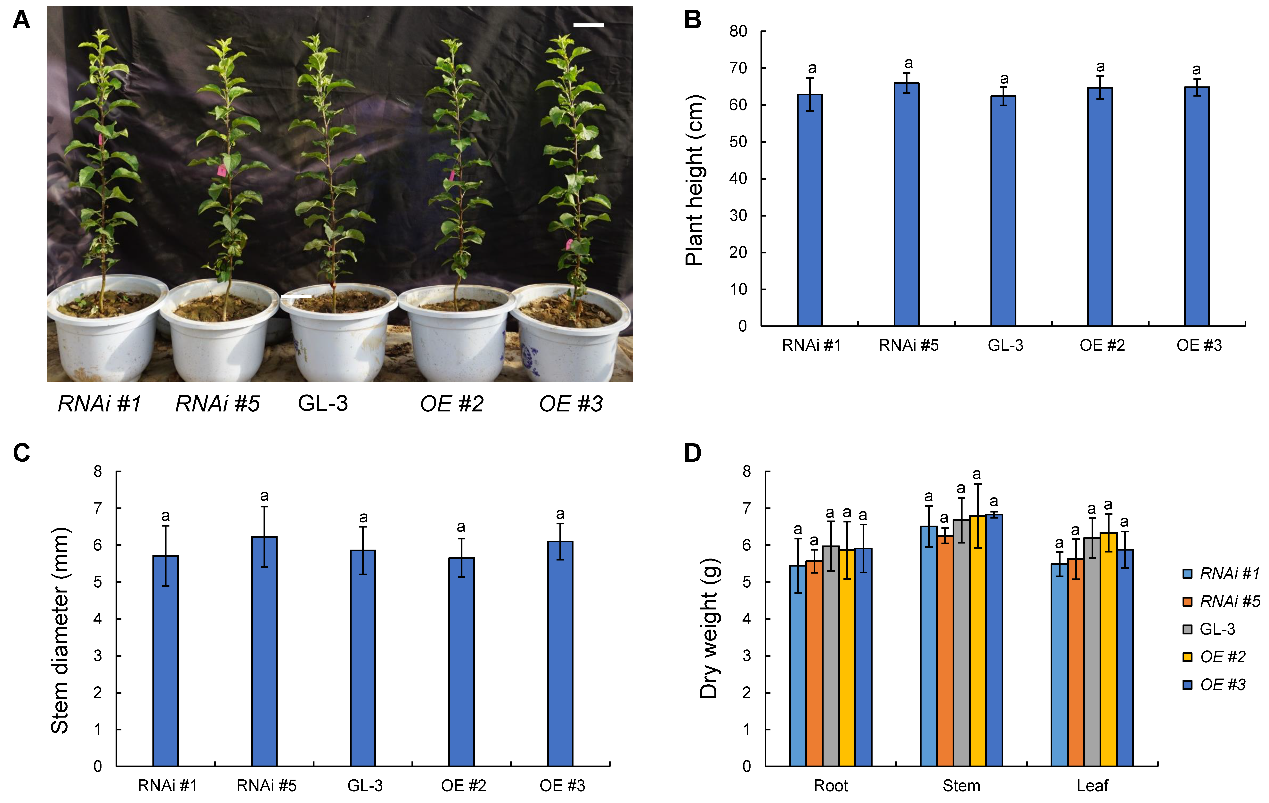


(**A**) Pictures of GL-3 and transgenic apple plants subjected to long-term moderate water deficit for 0 d. RNAi and OE denote *MdHB-7* RNAi and *MdHB-7* overexpressing apple plants, respectively. Scale bars, 10 cm. (**B**) The plant height of GL-3 and *MdHB-7* transgenic apple plants. (**C**) The stem diameter of GL-3 and *MdHB-7* transgenic apple plants. (**D**) The dry weight of GL-3 and *MdHB-7* transgenic apple plants. Data are presented as means ± SD (*n* = 20 for b and c, 20 plants from each line; *n* = 3 for d). Different letters indicate significant differences based on one-way ANOVA and Tukey's multiple range test (p < 0.05).

**Figure S2.** The plant height and stem diameter of GL-3 and *MdHB-7* transgenic apple plants during long-term moderate water deficit.


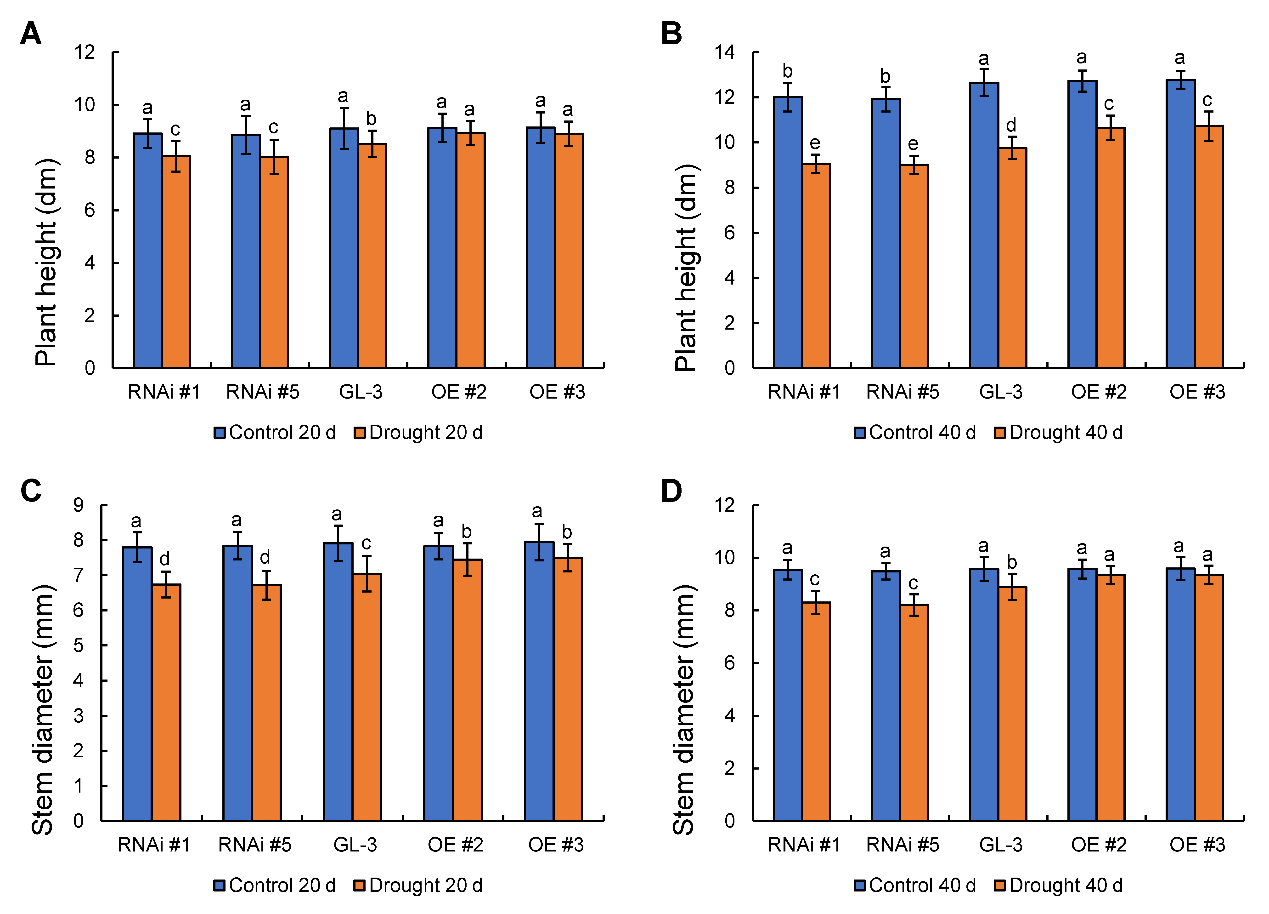


(**A**) The plant height of GL-3 and *MdHB-7* transgenic apple plants subjected to long-term moderate water deficit for 20 d. (**B**) The plant height of GL-3 and *MdHB-7* transgenic apple plants subjected to long-term moderate water deficit for 40 d. (**C**) The stem diameter of GL-3 and *MdHB-7* transgenic apple plants subjected to long-term moderate water deficit for 20 d. (**D**) The stem diameter of GL-3 and *MdHB-7* transgenic apple plants subjected to long-term moderate water deficit for 40 d. Data are presented as means ± SD (*n* = 20, 20 plants from each line). Different letters indicate significant differences based on one-way ANOVA and Tukey's multiple range test (*p* < 0.05).

**Figure S3.** Expression of *MdHB7-like*, *MdHD-Zip6* and *MdHD-Zip7* genes in GL-3 and transgenic apple lines.


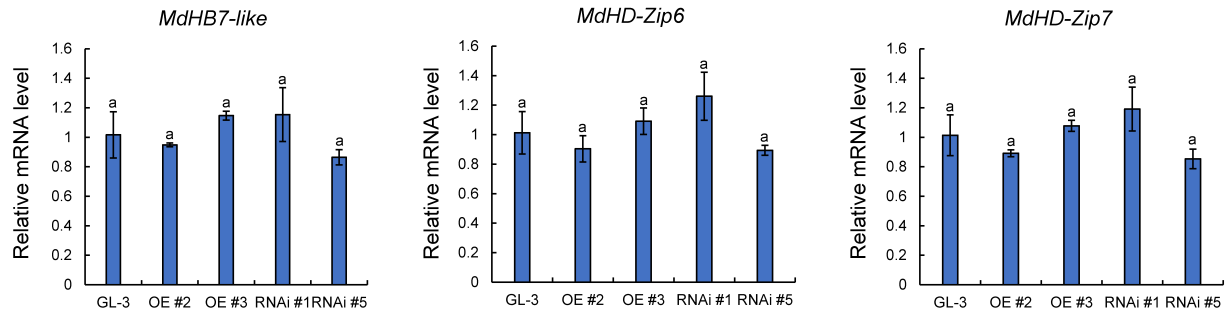


Data are presented as means ± SD (*n* = 3). Different letters indicate significant differences based on one-way ANOVA and Tukey's multiple range test (*p* < 0.05).

**Table S1** Application of primers and sequences

| Use | Primer name | Forward primer (5'- 3') | Reverse primer (5'- 3') |
| --- | --- | --- | --- |
| qRT-PCR | *qRT-MdHB7-like* | TGGAGCGGAGAGGAGTCATAGATTC | TGAGGTGTCGCAGAGGCATCC |
|  | *qRT-MdHD-Zip6-* | TCACCGGGAGCTGCTACTACAAG | GCCTCGACTCAGACTCAAAGATGG |
|  | *qRT-MdHD-Zip7* | CAGACGGCAGATGGATCGAGTTAC | GGCTTGTATGTGAATCTATGGCTCCTC |
|  | *qRT-MdEPF1* | CGCATAGCTCGGCCTCATT | CGACCCTGCCACTTGGACT |
|  | *qRT-MdEPF2* | TACGTGTTTGGCCTCCTCA | CCTCGATCCGCTTTGGTAT |
|  | *MdMDH* | CGTGATTGGGTACTTGGAAC | TGGCAAGTGACTGGGAATGA |
